# Effects of BCAA supplementation on plasma and mare's milk amino acid contents in *Yili* mares and growth performance of suckling foals

**DOI:** 10.3389/fvets.2025.1602363

**Published:** 2025-05-26

**Authors:** Xiang Ren, Yuheng Xue, Zhehong Shen, Xiaotian Liu, Xiaokang Chang, Jun Meng, Wanlu Ren, Jianwen Wang, Xinkui Yao, Yaqi Zeng

**Affiliations:** ^1^Xinjiang Horse Industry Association, Urumqi, China; ^2^School of Animal Science, Xinjiang Agricultural University, Urumqi, China

**Keywords:** branched-chain amino acids, Yili mare, free amino acids, suckling foal, growth performance

## Abstract

Branched-chain amino acids (BCAAs) play a crucial role in regulating nutritional metabolism in lactating animals. However, limited research has been conducted on BCAAs in equines. This study aimed to investigate the effects of different doses of BCAA supplementation on plasma and milk amino acid profiles in Yili mares, as well as the growth performance of their suckling foals, thereby providing a scientific basis for optimizing feeding management practices. Eighteen pairs of Yili mares and their sucklings were randomly assigned to four groups: a control group (Group D, no BCAA supplementation) and three experimental groups (S1, S2, and S3, receiving 38 g/day, 76 g/day, and 114 g/day of BCAA supplementation, respectively). The trial lasted for 67 days. The concentrations of 22 amino acids in plasma and milk were quantified using liquid chromatography-mass spectrometry (LC-MS), and their correlations with the body height, length, and weight of the foals were analyzed using SPSS software (one-way analysis of variance and Pearson correlation test). In mare plasma amino acids, the serine (Ser) content in group S1 was significantly higher than that in group D (*p* < 0.05). Additionally, in group S3, tryptophan (Trp), histidine (His), and aspartic acid (Asp) contents were markedly elevated. For mare milk amino acids, Ser content in group S1 was extremely significantly higher than in group D (*p* < 0.01), while aspartic acid (Asp) and alanine (Ala) contents were significantly increased in group S3. Regarding foal growth performance, body weight in group S3 was significantly greater than in group D. Moreover, group S2 exhibited superior trends in body height and length growth. Correlation analysis demonstrated that plasma Ser and creatine (Cr) were positively correlated with mare milk Ser and Cr. Mare milk threonine (Thr) showed a positive correlation with foal body height and length. Studies indicate that branched-chain amino acids (BCAA) regulate protein synthesis and amino acid metabolism via the mTOR pathway. In this experiment, 38 g/d BCAA enhanced mammary gland Ser transport, thereby increasing its content. Furthermore, 114 g/d BCAA promoted Asp and Ala accumulation, likely due to enhanced catabolic activity. The positive correlation between mare milk Thr, His, and skeletal development suggests that BCAA indirectly promotes growth through milk composition regulation. However, given the small sample size of this study, long-term validation is necessary.

## 1 Introduction

**Figure 1:**
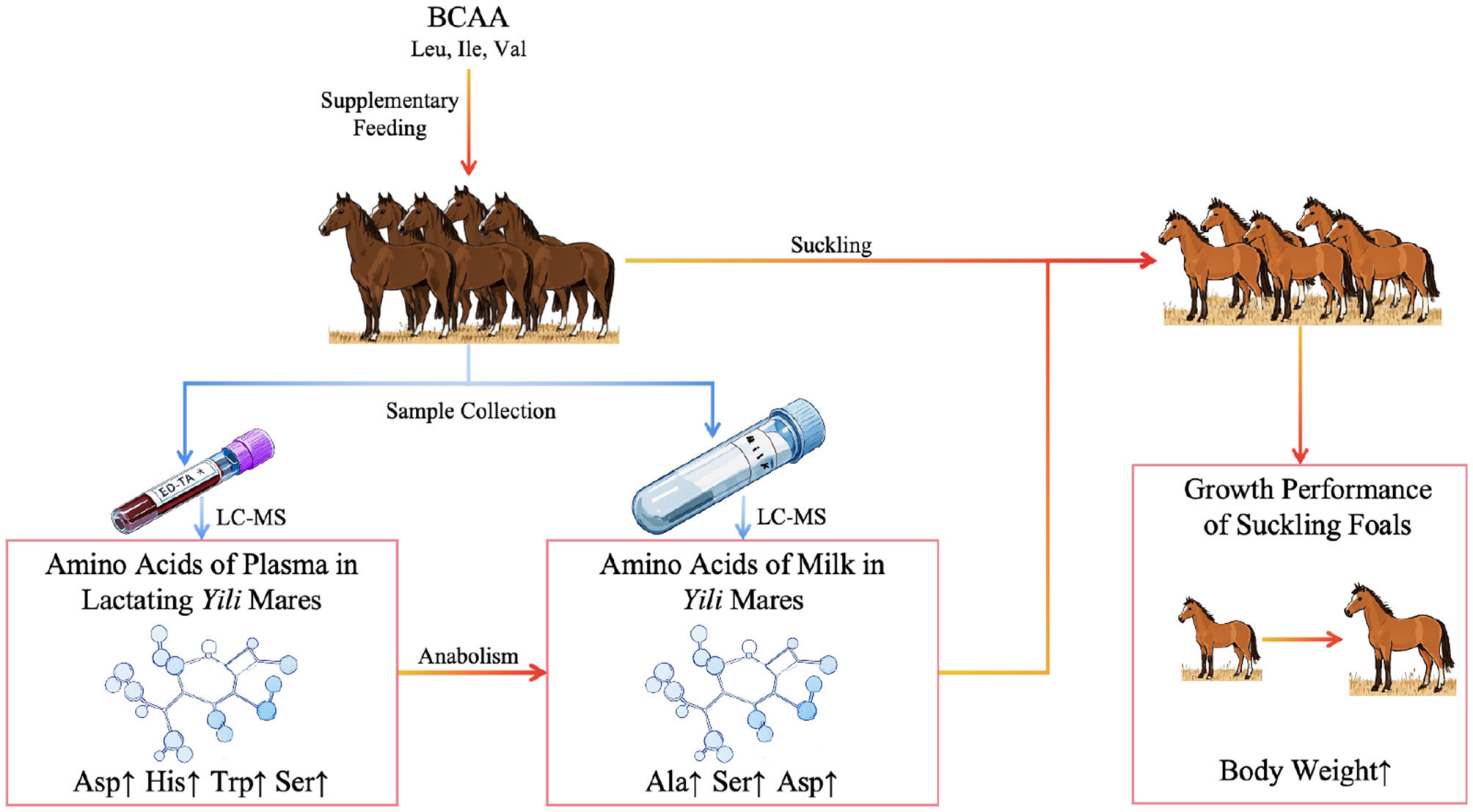
Schematic overview of the experimental design.

Amino acids, the fundamental building blocks of proteins, play indispensable roles in governing physiological processes critical to animal growth, development, and reproductive success. Recent advancements in maternal amino acid supplementation strategies have expanded research frontiers in lactation modulation and offspring vitality enhancement (1–3). Lactating mares face exceptional metabolic demands to sustain both physiological homeostasis and milk synthesis, with nutritional status directly determining mare's milk compositional quality. Branched-chain amino acids (BCAA)—leucine (Leu), isoleucine (Ile), and valine (Val)—constitute a distinctive category of essential amino acids that have attracted significant research attention for their regulatory functions in animal nutrient metabolism. Emerging evidence underscores BCAA's lactogenic potential in livestock production systems. Hultquist and Casper (4) demonstrated that Val supplementation enhances milk yield in late-lactation dairy cattle. At the cellular level, Gao et al. (5) revealed elevated Ile concentrations in bovine mammary epithelial cells stimulate sterol regulatory element-binding protein 1 (SREBP1) expression, thereby promoting lipogenesis. Complementary findings by Wang (6) identified a positive correlation between graded Leu supplementation in periparturient sow diets and progressive increases in milk lipid content. Most of the existing studies on BCAA in livestock production focus on animals such as cattle and pigs, and there are few studies on the specific effects of BCAA on the performance of equine animals. Especially in the feeding and management of mares during lactation, the effects of supplemental feeding of BCAA on the content of amino acids in plasma and milk of mares are still unclear. While existing studies have focused on the direct association between the high efficiency of mammary BCAA metabolism and milk component synthesis in ruminants, research on BCAA metabolism in horses is seriously lagging behind. This study systematically evaluates dose-dependent responses of plasma and mare's milk amino acid profiles to dietary BCAA supplementation, while establishing correlation matrices to decipher mammary amino acid utilization patterns (Figure 1). These findings are anticipated to provide theoretical foundations for optimizing feeding strategies and enhancing mare's milk quality in this economically vital equine population.

## 2 Materials and methods

### 2.1 Materials

The experiment utilized branched-chain amino acids (BCAA) obtained from Xinjiang Lianying Biotechnology Co., LTD. Following the amino acid requirement study for equine animals (7), the ratio of Isoleucine (Ile), Leucine (Leu), and Valine (Val) fed in the experiment was 1:2:1.2. The resulting mixture appeared as a white powder after thorough blending.

### 2.2 Design of experiments

The trial was conducted at *Zhaosu* Stud Farm in *Yili Kazak* Autonomous Prefecture, Xinjiang Uygur Autonomous Region, from July to September 2023. Eighteen lactating mares (body weight: 392.90 kg ± 12.18 kg) and their suckling foals were randomly divided into four groups: a control group (Group D, *n* = 4) and three treatment groups (S1, S2, S3; *n* = 5, 5, 4, respectively). All mares were managed under identical feeding conditions for 67 d (7-d adaptation period and 60-d experimental phase). Each mare received 2 kg/d of concentrate supplement (composition and nutritional levels detailed in Table 1). Groups S1, S2, and S3 were additionally supplemented with 38, 76, and 114 g/d branched-chain amino acid (BCAA), respectively.

**Table 1:** Nutrient level of concentrate supplements for Yili mares during lactation (dry matter basis) %.

**Feed composition**	**Content**	**Nutritional level**	**Content**
Maize	46	Dry matter	90.76
Barley	8	Crude protein	14.44
Wheat bran	8	Neutral detergent fiber	61.68
Rape-seed meal	28	Acid detergent fiber	9.95
Premix	10	Calcium (Ca)	0.63
Total	100	Phosphorous (P)	0.35

Each kg premix was supplied with 20,000 IU of VA1, 30,000 IU of VD3, 300 mg of VB3, 2,500 mg of VE, 250 mg of Cu, 1,200 mg of Fe, 1,200 mg of Zn, 1,100 mg of Mn, 8 mg of I, 6 mg of Se, and 4 mg of Co.

### 2.3 Feeding management

During the trial, mares and foals were moved daily at 10:00 h from pasture to the milking parlor. Following foal separation, mares were guided to individual stalls and supplemented with branched-chain amino acid (BCAA) at respective doses mixed into 2 kg of concentrate. Milking was performed every 1.5 h (four times/d). After the final milking session, mares and foals were returned to pasture for unrestricted grazing.

### 2.4 Sample collection and determination

#### 2.4.1 Blood samples were collected from mares

On the 60th day of the experiment, mares were fasted for 2 h before blood samples were collected using EDTA anticoagulant. Specifically, 10 mL of blood was drawn from the jugular vein to obtain plasma, which was subsequently centrifuged at 3,000 r/min for 15 min. The resulting supernatant was then transferred to frozen storage tubes, promptly frozen in liquid nitrogen for 15 min, and finally stored in a refrigerator at −80°C for the analysis of free amino acid content.

#### 2.4.2 Mare milk sample collection

Milk samples were collected on day 60 of the experiment and manually milked every 1.5 h (at 11:00, 12:30, 14:00, and 15:30, respectively). Subsequently, 25 mL from each of the four milk samples were combined, with 5 mL aliquoted into frozen tubes for rapid freezing in liquid nitrogen for 15 min, followed by storage at −80°C in a refrigerator. The free amino acid content in the milk was assessed using the identical determination index employed for plasma free amino acids.

#### 2.4.3 Determination of free amino acid content

The detection was carried out by Beijing Novogene Technology Co., Ltd. using high performance liquid chromatography tandem mass spectrometry (LC-MS) for quantitative analysis. The measured indexes include: Glycine (Gly), Serine (Ser), Methionine (Met), Proline (Pro), Leucine (Leu), Creatine (Cr), Glutamic (Glu), Phenylalanine (Phe), Lysine (Lys), Arginine (Arg), Tryptophan (Trp), Tyrosine (Tyr), Histidine (His), Valine (Val), Ornithine (Orn), Alanine (Ala), Isoleucine (Ile), Aspartic (Asp), Threonine (Thr), Taurine (Tau), glutamine (gln), asparaginate (asn).

#### 2.4.4 Measurement of growth performance in suckling foals

Foals were restrained for measurement at 0 d and 60 d. Withers height, body length, chest girth, cannon bone circumference, and body weight were measured using a measuring stick and tape.

### 2.5 Data processing

Data were initially organized in Excel and analyzed using SPSS 26.0. One-way ANOVA with Duncan's multiple-range test was applied, with significance thresholds set at *p* < 0.05 (significant) and *p* < 0.01 (highly significant). Results are expressed as mean ± SD. To investigate correlations between plasma and mare's milk free amino acid content in Yili mares, as well as associations between milk amino acids and foal growth performance, Pearson correlation analysis was conducted in SPSS 21.0 using data from day 60. Figures were generated using Excel and the MetWare Cloud platform.

## 3 Results

### 3.1 Effects of BCAA supplementation on plasma free amino acid content of *Yili* mares

**Figure 2:**
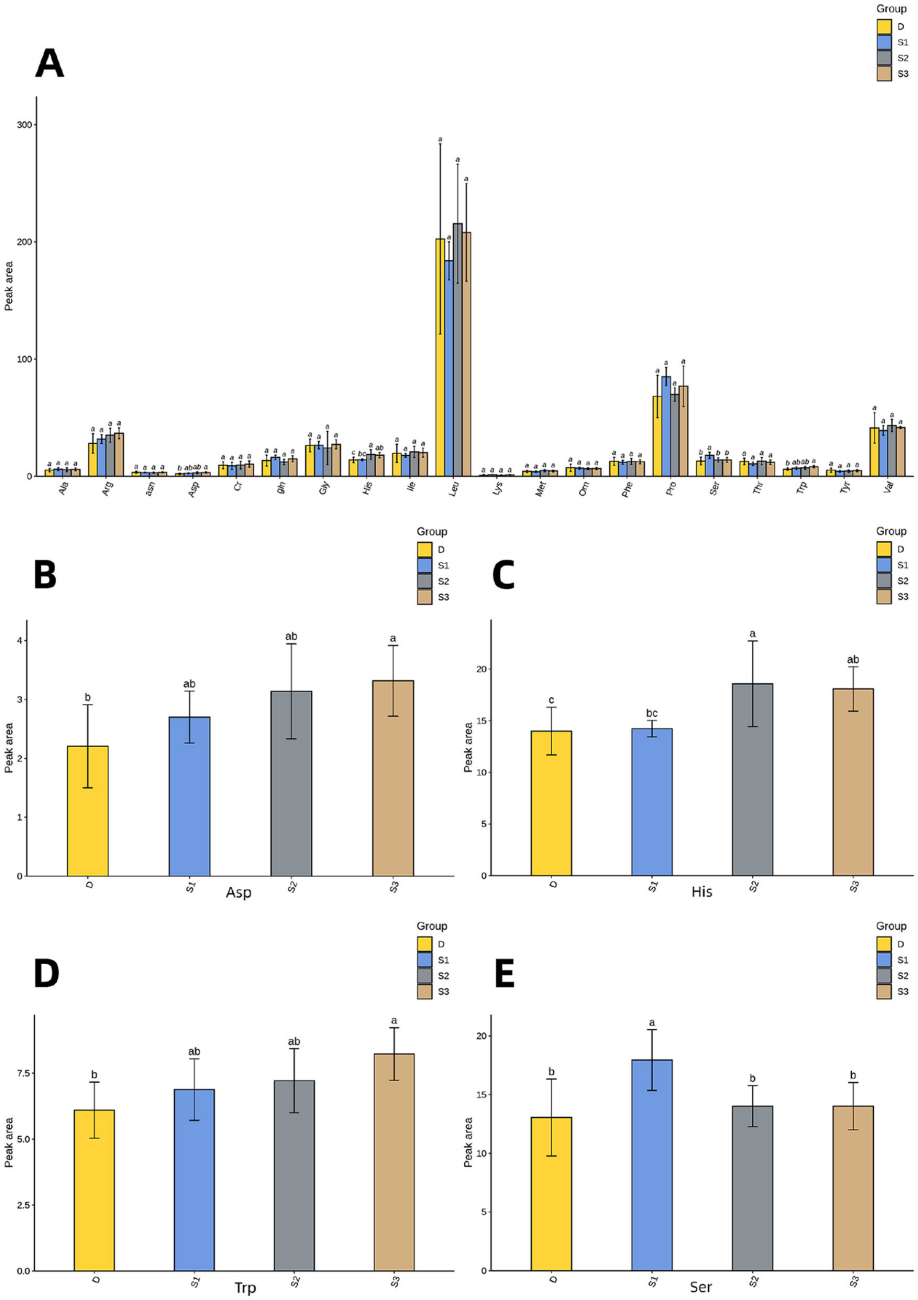
For all the letters represented in the bar graphs, different lowercase letters indicate *p* < 0.05 **(A)** Histogram of plasma amino acid metabolomics quantitative results in *Yili* mares. **(B)** Histogram of Asp quantification results. **(C)** Histogram of His quantification results. **(D)** Histogram of Trp quantification results. **(E)** Histogram of Ser quantification results.

Figure 2 demonstrates that histogram of the quantitative results of plasma amino acid metabolome in mares. The plasma Asp concentrations in group S3 significantly exceeded those in D (*p* < 0.05, Figure 2B). Both S2 and S3 demonstrated higher His content than D (*p* < 0.05, Figure 2C), with S2 additionally surpassing S1 in His concentrations (*p* < 0.05). The Trp content in group S3 was significantly higher compared to that in group D (*p* < 0.05, Figure 2D), while showing comparable values to S1 and S2 (*p* > 0.05). Notably, Ser levels in groups D, S2, and S3 were significantly lower than those in S1 (*p* < 0.05, Figure 2E), with no significant differences detected among D, S2, and S3 (*p* > 0.05, Table 2).

**Table 2:** Effects of BCAA supplementation on plasma free amino acid content of *Yili* mares.

**Amino acid**	**Free amino acid content in plasma (**μ**g/mL)**			
	**D**	**S1**	**S2**	**S3**
Gly	26.40 ± 5.46	26.56 ± 3.23	24.19 ± 14.30	27.26 ± 3.91
Ser	13.05 ± 3.28^b^	17.94 ± 2.59^a^	14.01 ± 1.75^b^	14.02 ± 2.01^b^
Met	4.25 ± 0.98	4.02 ± 0.51	4.96 ± 0.87	4.66 ± 0.68
Pro	68.17 ± 18.00	85.10 ± 7.73	69.74 ± 5.79	76.91 ± 17.29
Leu	202.53 ± 81.15	183.87 ± 16.19	215.68 ± 50.76	208.16 ± 41.63
Cr	9.50 ± 2.94	9.00 ± 2.92	9.62 ± 3.19	10.46 ± 2.75
Phe	12.80 ± 3.35	11.99 ± 1.65	12.66 ± 2.60	12.47 ± 1.63
Lys	0.98 ± 0.59	1.34 ± 0.13	0.93 ± 0.42	1.25 ± 0.22
Arg	28.07 ± 8.41	31.77 ± 3.87	35.04 ± 6.00	36.78 ± 4.61
Trp	6.10 ± 1.06^b^	6.88 ± 1.17^ab^	7.22 ± 1.22^ab^	8.23 ± 1.00^a^
Tyr	5.29 ± 1.68	4.27 ± 0.55	4.56 ± 1.00	4.9 ± 0.87
His	14.00 ± 2.31^c^	14.23 ± 0.79^bc^	18.57 ± 4.15^a^	18.08 ± 2.15^ab^
Val	41.32 ± 13.07	39.10 ± 4.20	43.37 ± 5.21	41.61 ± 0.91
Orn	7.37 ± 3.08	6.96 ± 0.99	6.53 ± 0.76	6.65 ± 1.02
Ala	5.32 ± 1.30	6.17 ± 1.25	5.74 ± 1.60	5.91 ± 1.08
Ile	19.60 ± 7.75	17.88 ± 1.67	20.84 ± 4.78	20.21 ± 3.87
Asp	2.21 ± 0.71^b^	2.7 ± 0.44^ab^	3.14 ± 0.81^ab^	3.32 ± 0.60^a^
Thr	12.69 ± 2.82	10.75 ± 1.50	12.96 ± 3.03	12.00 ± 2.01
gln	13.48 ± 4.63	16.17 ± 1.92	12.28 ± 2.46	15.00 ± 2.58
asn	3.51 ± 0.84	3.28 ± 0.35	3.14 ± 0.56	3.39 ± 0.51

No letter or the same letter in peer data shoulder indicates no significant difference (P > 0.05), and different lowercase letters indicate significant difference (p < 0.05).

### 3.2 Effect of BCAA supplementation on free amino acid content in *Yili* mare's milk

**Figure 3:**
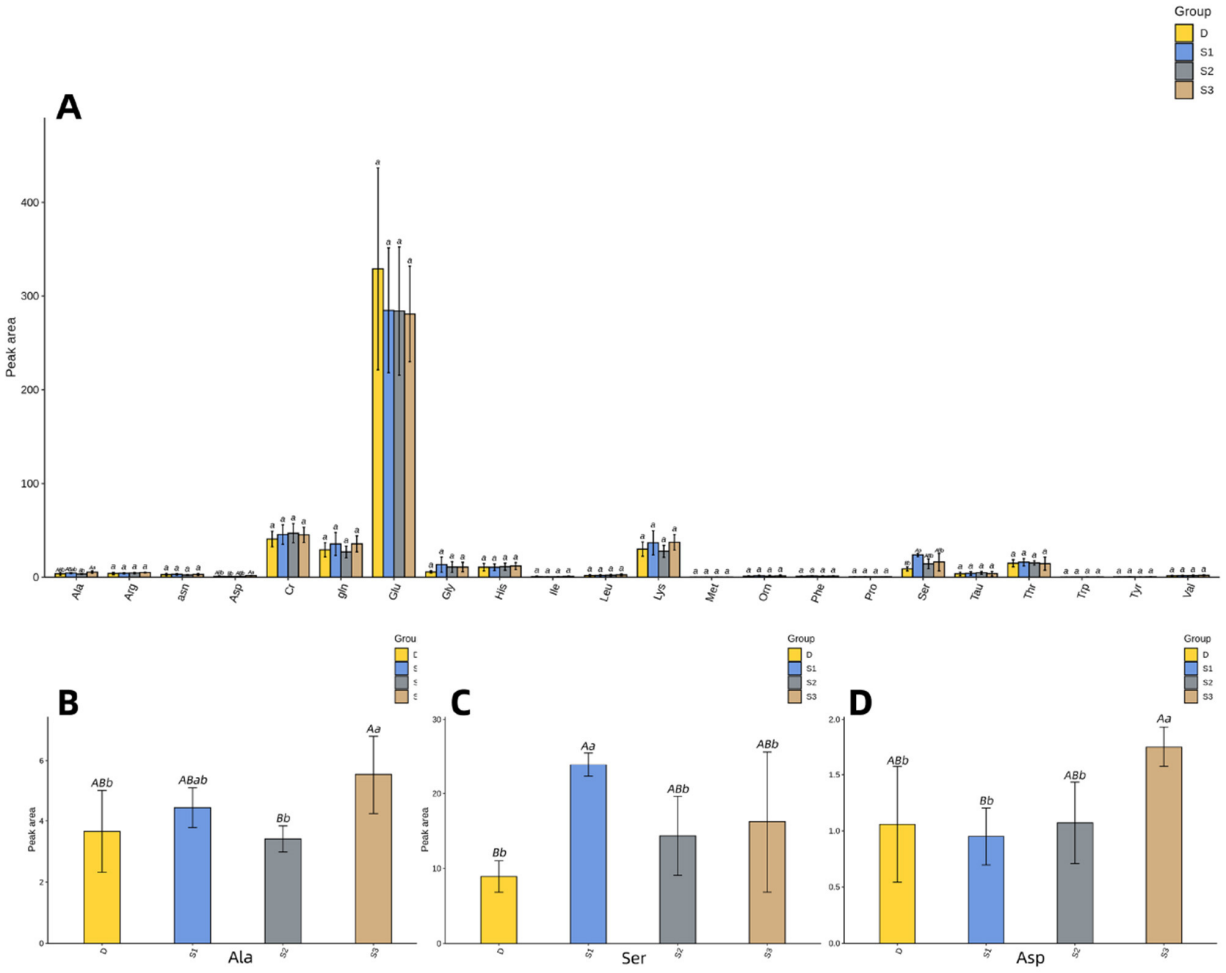
For all the letters represented in the bar graph, different lowercase letters indicate *p* < 0.05, different capital letters indicate *p* < 0.01. **(A)** Histogram of quantitative results of amino acid metabolomics in mare's milk. **(B)** Histogram of Ala quantification results. **(C)** Histogram of Ser quantification results. **(D)** Histogram of Asp quantification results.

Figure 3 demonstrates that histogram of the quantitative results of the amino acid metabolome of *Yili* mare's milk. Figure 3B illustrates that group S3 exhibited elevated Ala concentrations compared to both D and S1 (*p* < 0.05), while demonstrating a more pronounced increase relative to S2 (*p* < 0.01) in mare's milk. The Ser concentrations in groups D, S2, and S3 were significantly lower than those in S1 (*p* < 0.05, Figure 3C), with no significant differences observed among D, S2, and S3 (*p* > 0.05, Table 3). Notably, Ser levels in S1 were substantially higher than in D (*p* < 0.01, Figure 3C). Similarly, Asp levels in S3 surpassed those in D and S2 (*p* < 0.05), with a highly significant difference compared to S1 (*p* < 0.01, Figure 3D).

**Table 3:** Effects of BCAA supplementation on the content of free amino acids in *Yili* mare's milk.

**Amino acid**	**Free amino acid content in mare's milk (**μ**g/mL)**			
	**D**	**S1**	**S2**	**S3**
Gly	5.67 ± 1.26	13.59 ± 8.10	10.95 ± 5.66	10.88 ± 5.01
Ser	8.91 ± 2.10^Bb^	23.93 ± 1.61^Aa^	14.35 ± 5.27^ABb^	16.23 ± 9.41^ABb^
Met	0.24 ± 0.03	0.32 ± 0.11	0.29 ± 0.14	0.22 ± 0.02
Pro	0.60 ± 0.20	0.66 ± 0.19	0.58 ± 0.08	0.67 ± 0.10
Leu	1.88 ± 0.89	2.03 ± 0.69	2.24 ± 0.79	2.58 ± 0.86
Cr	40.80 ± 8.30	45.62 ± 10.35	47.05 ± 10.20	45.23 ± 8.11
Glu	328.90 ± 107.72	284.76 ± 66.70	283.98 ± 68.36	280.87 ± 51.00
Phe	1.15 ± 0.35	1.44 ± 0.21	1.20 ± 0.41	1.36 ± 0.29
Lys	30.17 ± 7.60	36.76 ± 12.91	27.78 ± 6.42	37.40 ± 8.24
Arg	4.03 ± 1.30	4.26 ± 0.69	4.47 ± 0.84	5.01 ± 0.48
Trp	0.29 ± 0.09	0.39 ± 0.04	0.35 ± 0.18	0.39 ± 0.14
Tyr	0.52 ± 0.09	0.63 ± 0.18	0.43 ± 0.18	0.59 ± 0.29
His	10.78 ± 3.92	10.72 ± 3.54	11.25 ± 3.84	11.93 ± 3.68
Val	1.65 ± 0.45	1.77 ± 0.43	1.87 ± 0.54	2.13 ± 0.45
Orn	1.22 ± 0.41	1.62 ± 0.59	1.25 ± 0.90	1.46 ± 1.02
Ala	3.66 ± 1.34^ABb^	4.43 ± 0.65^ABab^	3.41 ± 0.42^Bb^	5.52 ± 1.28^Aa^
Tau	3.62 ± 1.83	4.09 ± 1.88	4.75 ± 1.54	3.82 ± 2.36
Ile	0.79 ± 0.56	0.66 ± 0.18	0.72 ± 0.23	1.08 ± 0.33
Asp	1.06 ± 0.51^ABb^	0.95 ± 0.25^Bb^	1.07 ± 0.36^ABb^	1.75 ± 0.18^Aa^
Thr	14.97 ± 3.85	16.02 ± 3.98	15.29 ± 2.40	14.54 ± 7.06
gln	29.40 ± 7.44	35.54 ± 12.23	27.09 ± 6.25	35.65 ± 8.52
asn	2.85 ± 1.19	3.11 ± 0.73	2.46 ± 0.32	2.99 ± 0.94

No letter or the same letter in peer data shoulder indicates no significant difference (*P* > 0.05), and different lowercase letters indicate significant difference (*p* < 0.05); Different capital letters indicate very significant differences (*p* < 0.01), as shown in the following table.

### 3.3 Effects of BCAA supplementation in lactating Yili mares on growth performance of suckling foals

**Figure 4:**
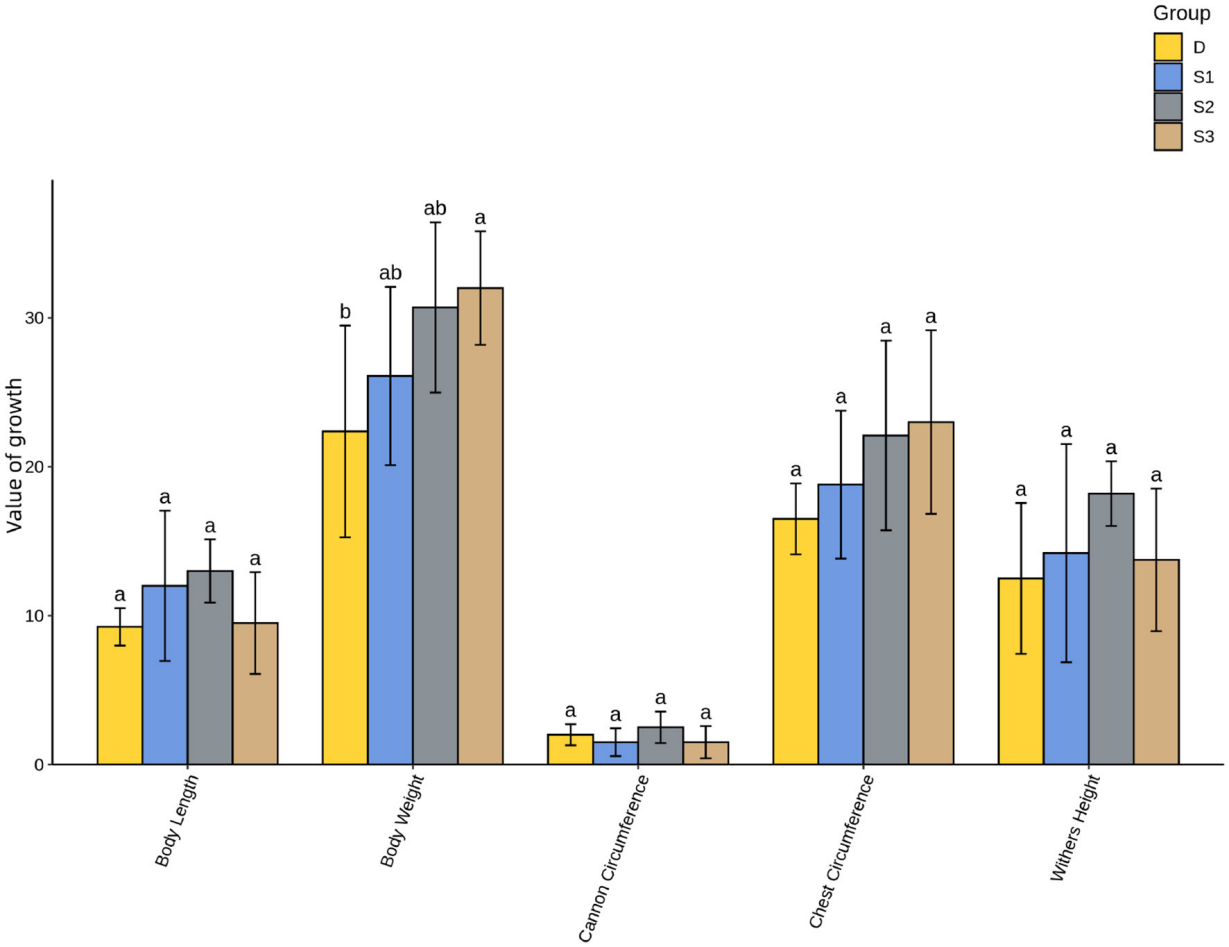
Histogram of One-Way ANOVA analysis for growth performance in suckling foals. For all the letters represented in the bar graph, different lowercase letters indicate *p* < 0.05.

As shown in Figure 4, suckling foals in Group S3 exhibited significantly higher body weight gain compared to Group D (*p* < 0.05). Chest circumference changes followed a similar trend across groups as body weight gain, though no significant differences were observed between treatment groups and the control (*p* > 0.05). No significant differences were detected in withers height, body length, or cannon bone circumference gains between experimental groups and the control (*p* > 0.05). However, similar trends emerged, with Group S2 demonstrating higher mean growth values than Groups D, S1, and S3.

### 3.4 Correlation analysis between free amino acids in mare plasma and free amino acids in milk

**Figure 5:**
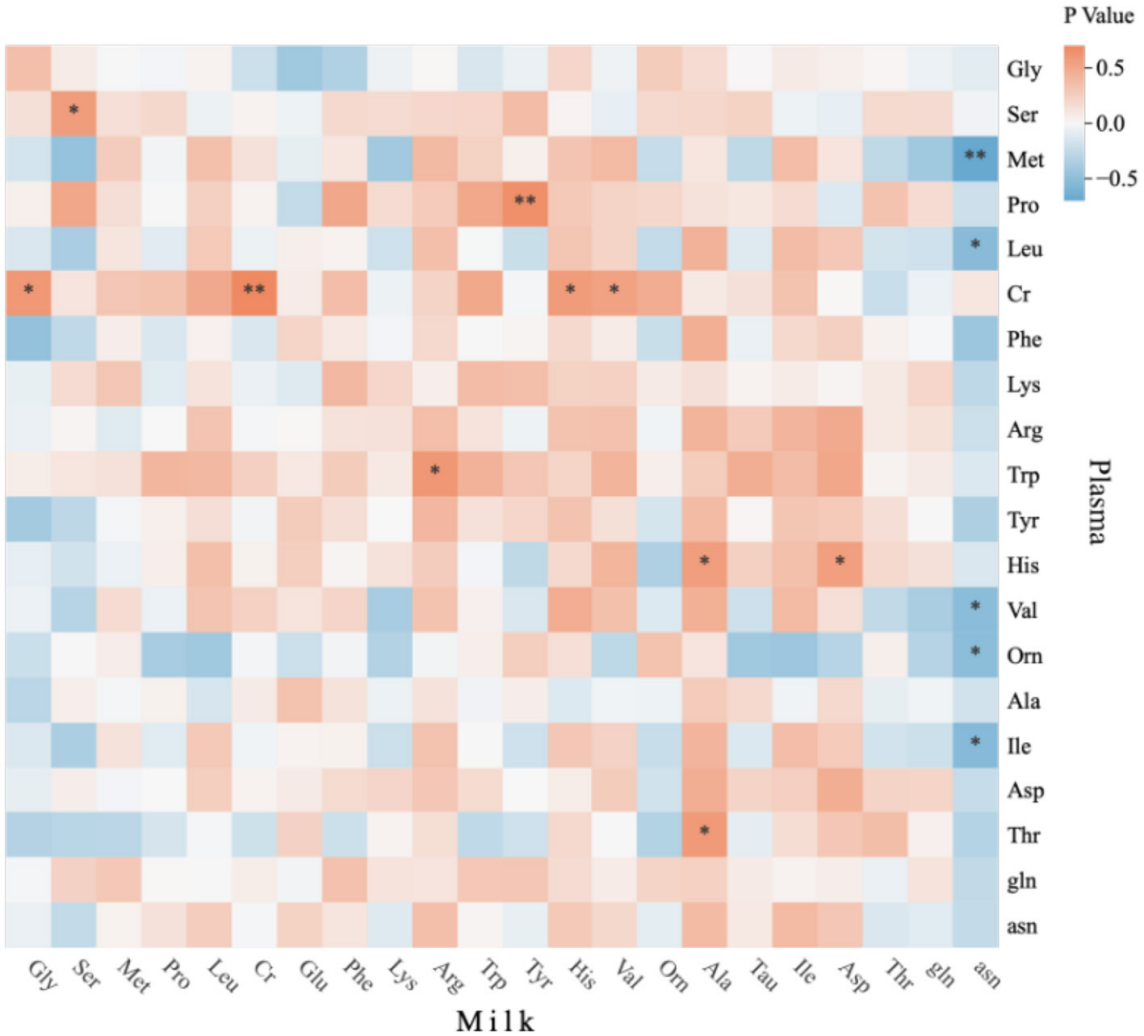
Heat map of correlation between amino acids in mare plasma and milk of *Yili* mares. * *p* < 0.05, ** *p* < 0.01.

Figure 5 illustrates correlations between plasma and milk amino acid content in lactating *Yili* horses. Plasma BCAA exhibits a positive but non-significant correlation with milk BCAA (*p* > 0.05). The figure further reveal significant positive correlations between plasma and milk Ser (*p* < 0.05) and Cr (*p* < 0.01). Plasma Cr demonstrates positive associations with milk Gly, His, and Val (*p* < 0.05), while plasma His correlates positively with milk Ala and Asp (*p* < 0.05). Notably, milk asn demonstrates a negative correlation with plasma BCAA (*p* < 0.05). Milk Val shows a positive association with plasma Cr (*p* < 0.05), and milk Arg correlates with plasma Trp (*p* < 0.05). The figure highlight a highly significant positive correlation between milk Tyr and plasma Pro (*p* < 0.01), alongside a positive relationship between milk Ala and plasma Thr (*p* < 0.05).

### 3.5 Correlation analysis between amino acids in mare's milk and growth performance of suckling foals

**Figure 6:**
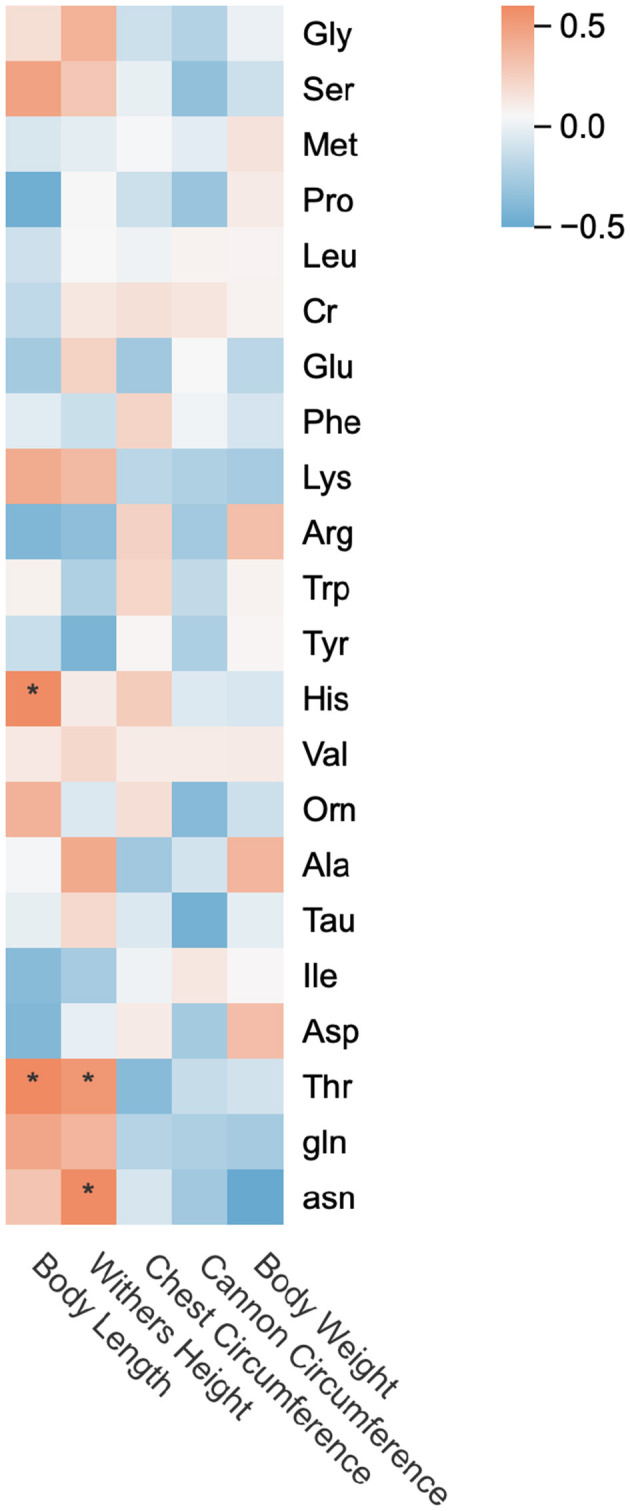
Heat map of correlation between amino acids in mare's milk and growth performance of suckling foals. * *p* < 0.05, ** *p* < 0.01.

As shown in Figure 6, changes in Thr content in mare's milk exhibited significant positive correlations (*p* < 0.05) with growth changes in body height and body length of suckling foals. Body length growth changes were significantly positively correlated with His content variations in milk (*p* < 0.05), while body height growth changes showed a significant positive correlation with asn content fluctuations (*p* < 0.05). Chest circumference and body weight growth changes demonstrated positive correlations with Arp and Asp content in milk, and body weight changes additionally correlated with Ala content. However, none of these correlations reached statistical significance (*p* > 0.05).

## 4 Discussion

### 4.1 Effect of BCAA supplementation on plasma amino acid content in *Yili* mares

BCAA, as essential amino acids for animals, can provide amino groups for the synthesis of Asp and gln (8). In this experiment, after BCAA supplementation in mares, the Asp content in Group S3 was significantly higher than in Group D, while gln level in Group S1 showed an increasing trend compared to Group D. This may be due to the addition of appropriate BCAA quantities, which promoted relevant metabolic pathways in the mare's body, thereby inducing changes conducive to Asp accumulation in plasma. McGuire et al. (9) found that amino acids are preferentially absorbed and utilized by the mammary gland through oxidation or incorporation into milk proteins. Griinari et al. (10) showed that insulin (INS) can inhibit protein degradation in muscles, and reduced protein turnover decreases circulating amino acid concentrations, affecting free amino acid levels in blood. In this study, the average BCAA content in Group S1 was lower than in Group D, possibly due to increased INS levels from concentrate supplementation, which enhanced BCAA utilization along the feeding gradient, thereby accelerating the mammary gland's absorption rate of free amino acids for milk protein synthesis. BCAA metabolism can influence the concentrations of other amino acids, including Ser (11), α-ketoglutarate (α-KG) produced by BCAA decomposition is not only an intermediate in the tricarboxylic acid (TCA) cycle, but also indirectly regulates the supply of 3-phosphoglycerate (3-PG), a substrate synthesized by Ser. In addition, branchedchain α-keto acids (BCKAs) may increase the production of 3-PG by activating pyruvate dehydrogenase kinase (PDK) to inhibit pyruvate dehydrogenase (PDH) and promote carbon flow to gluconeogenesis (12). In this experiment, Ser content in Group S1 was significantly higher than in Groups D, S2, and S3, indicating optimal BCAA conversion and utilization in lactating mares at a BCAA feeding rate of 38 g/d under these experimental conditions. BCAA directly activates mechanistic target of rapamycin C1 (mTORC1), which promotes protein synthesis by phosphorylating S6K1 and inhibiting 4E-BP1 (13). Studies have shown that mTORC1 can also up-regulate the expression of ATF4, which is a transcriptional activator of key SSP enzymes (PHGDH, PSAT1) (14). Thus, BCAA may drive serine synthesis via the mTORC1-ATF4 axis. BCAA supplementation may increase plasma BCAA concentrations, thereby affecting other amino acids such as His and Asp. In this experiment, His and Asp levels in Group S3 were higher than in Group D, consistent with the findings of Pitkänen et al. (15).

### 4.2 Effect of BCAA supplementation on mare's milk amino acid content

Free amino acids in milk can be directly absorbed by the body, and dietary amino acid supplementation can improve milk amino acid composition. BCAA are major components of milk proteins. Wei et al. (16) demonstrated that adding BCAA to dairy cow diets increases BCAA content in milk. In this study, the average BCAA content in experimental groups was higher than in Group D, aligning with previous findings, though the difference was not statistically significant, possibly due to BCAA supplementation levels. High activities of asparagine synthetase and glutamine synthetase in mammary tissue provide biochemical mechanisms for synthesizing Asp, asn, Glu, and other non-essential amino acids from BCAA. As BCAA concentration increases, its degradation rate proportionally rises (17). In this experiment, Asp and Ala contents in mare's milk increased significantly at 114 g/d supplementation, while Ser content increased notably at 38 g/d, likely related to accelerated BCAA degradation in the mammary gland. Ser is one of the crucial amino acids in lactating mare's milk (18). In this experiment, Ser, Gly, and Met levels in Group S1′s milk were higher than in other groups, though Gly and Met showed no significant differences. This may occur because Ser participates in one-carbon metabolism and the Met cycle through enzymatic interconversion with Gly (19), leading to increased Ser uptake from plasma to the mammary gland. Additionally, Ser phosphorylation by Ser kinase plays a key role in Met and BCAA synthesis (20, 21), suggesting that 38 g/d BCAA supplementation enhances this amino acid metabolic pathway under experimental conditions. In Group S3, Ala and Asp levels were significantly higher than in Group D, indicating that BCAA supplementation improves mammary gland BCAA utilization efficiency, thereby affecting specific amino acid levels in mare's milk, consistent with Lei et al. (22). Protein dephosphorylation is a critical process in mammary milk synthesis (23), involving amino acids such as Ala, Asp, and Ser (24–26). In this experiment, Ala, Asp, and Ser levels in the treatment group's milk differed significantly from Group D, suggesting that appropriate BCAA quantities may promote anabolic pathways of these amino acids in mammary cells or enhance their uptake capacity, leading to milk composition changes. The mammary gland can convert plasma free amino acids into Tyr and others (27). In this study, Tyr and Met levels in milk were substantially lower than in plasma, indicating active mammary utilization and conversion of these amino acids.

### 4.3 Correlation between plasma and mare's milk amino acid content

In this study, plasma and milk Ser levels under 38 g/d BCAA supplementation were significantly higher than in Groups D, S1, and S2, potentially due to BCAA metabolism and transport promoting milk synthesis. Leu and Ile activate the mTOR signaling pathway to stimulate mammary epithelial cell proliferation and milk protein synthesis (28, 29), while the mTOR pathway critically regulates amino acid transporter expression (30). Correlation analysis revealed a significant positive correlation between plasma and milk Ser, likely reflecting increased mammary demand for amino acids during lactation, elevating transporter expression (31). Amino acid transport systems directly influence milk protein synthesis; for example, Asp, Gly, Pro, Ser, and Tyr exhibit turnover rates exceeding 50%, suggesting transporter-driven influx (32). Exogenous Ser supplementation can enhance antioxidant capacity in lactating sows and piglets via maternal transfer (33), implying potential for improving antioxidant capacity in *Yili* mares through Ser supplementation. This study found a significant positive correlation between plasma Cr and milk Cr, possibly through mammary epithelial uptake of plasma Cr via sodium/chloride-dependent transporters (CreaT) or oligopeptide transporters (e.g., PEPT1/2), followed by secretion into milk. Muscle Cr concentrations are nearly 200-fold higher than in plasma, indicating efficient transmembrane transport (34). Plasma Cr also positively correlated with milk Gly, His, and Val. Gly, His, and Val participate in Cr biosynthesis through distinct pathways: Ser serves as a Gly precursor (35), and Gly is essential for Cr synthesis (36), aligning with the plasma-milk Ser correlation and indicating maternal provision of Cr and Gly precursors via circulation. His is vital for hemoglobin synthesis (37), and Cr metabolism may influence His methylation via methyl group donation (e.g., 3-methylhistidine formation) (38), thereby regulating milk His levels. As a BCAA, Val shares metabolic pathways with Leu and Ile; plasma Cr may reduce BCAA catabolism by promoting muscle protein synthesis (39), increasing Val transfer into milk. Milk BCAA (e.g., Val) are crucial for foal muscle development (40), suggesting maternal metabolic prioritization of Val allocation to milk. Plasma His showed significant positive correlations with milk Ala and Asp, potentially through His transamination to Ala and Asp's role in urea cycle-mediated ammonia detoxification (41). Plasma Thr positively correlated with milk Ala, possibly via Thr-derived pyruvate and Gly serving as Ala precursors in the mammary gland, with Thr enhancing alanine synthesis through transaminase (GPT) activation or alanine dehydrogenase inhibition (42). Milk gln negatively correlated with plasma BCAA, Met, and Orn, likely due to competitive absorption via sodium-dependent neutral amino acid transporters (3). gln is hydrolyzed to Glu and ammonia, which enters the urea cycle to produce Orn (43). During lactation, mares prioritize gln for milk protein synthesis over urea cycle entry, reducing plasma Orn. Efficient mammary gln uptake may limit hepatic gln availability, suppressing urea cycle Orn production (43). gln serves as an Orn precursor for polyamine synthesis, which requires S-adenosylmethionine (SAM) derived from Met, indirectly depleting Met. gln also upregulates cystathionine-β-synthase (CBS) activity, promoting Met conversion to glutathione (GSH) and reducing plasma free Met (44), indicating urea cycle suppression and accelerated methyl donor metabolism.

### 4.4 Correlation between amino acid content of horse milk and growth performance of suckling foals

Foal body height and body length growth are related to skeletal development (45). Ser supplementation improves both femur length and bone mineral density in piglets, while significantly increasing body length growth rate (46). His deficiency reduces daily weight gain in weaned piglets (47), confirming the universal essentiality of Ser and His in young monogastric mammals. asn provides gln to osteoblasts through the SLC1A5 transporter, supporting energy metabolism required for their differentiation (48). Thr has been demonstrated to enhance growth rate and feed conversion efficiency in offspring (49), closely associated with its promotive effects on skeletal development. This study found positive correlations between changes in Ser, His, Thr, gln, and asn content in mare's milk and body length growth of suckling foals. Notably, variations in Ser, Thr, gln, and asn content also positively correlated with body height growth, indicating that BCAA supplementation in lactating mares alters milk amino acids influencing foal growth performance. These results suggest that BCAA supplementation in lactating mares may alter the amino acid content in horse milk and thus may have a positive effect on the growth performance of Suckling foals. Arg activates the mTOR pathway to promote muscle fiber hyperplasia and skeletal metabolism regulation (50, 51). Although changes in Arg and Asp content in mare's milk showed positive correlations with foal weight and chest circumference growth, these relationships were not statistically significant. Asp and its metabolite aspartate aminotransferase (KYNA) can promote osteoblast differentiation and bone formation by activating Wnt/β-catenin signaling pathway. Studies have shown that KYNA can improve bone mineral density in patients with osteoporosis and play a role by regulating osteoblast differentiation and bone remodeling (52). The specific impacts on foal growth performance require further investigation. The changes of Ala content in horse milk were positively correlated with the changes of body weight and body height of Suckling foals, but no significant correlation was found. Under the experimental conditions, BCAA supplementation at 76 g/d positively influenced body height and body length growth in suckling foals, while 114 g/d BCAA supplementation improved body weight and chest circumference development.

### 4.5 Limitations of the study

In this study, due to the limited number of Yili horses and the constraints of experimental resources, we selected 18 horses for the experiment; although this sample size has some reference value in the preliminary exploratory study, it does limit the universality and statistical significance of the study results. We also recognize that the 60-day trial period may not be sufficient to fully assess the long-term effects of BCAA supplementation, particularly in terms of bone development, immune function in foals, and long-term health and reproductive performance in mares. Future studies may consider extending the trial period to more fully evaluate the long-term effects of BCAA supplementation in lactating mares and provide a more comprehensive scientific basis for optimizing the nutritional management of *Yili* horses.

## 5 Conclusions

This study demonstrates that supplementing *Yili* mares with 38 g/d BCAA significantly increases plasma and milk Ser levels, with a significant positive correlation between plasma and milk Ser. Supplementation at 76 g/d significantly increased plasma His content in lactating *Yili* mares while positively influencing body height and body length development in suckling foals. At 114 g/d, supplementation significantly elevated plasma Asp and Trp levels, as well as Asp and Ala content in mare's milk, with concurrent positive effects on foal body weight and chest circumference growth. Future applications may utilize Ser as a dietary supplement for lactating Yili mares to enhance the nutritional value of mare's milk. Similarly, His and Thr could be targeted as supplements for suckling foals to improve growth performance. Further research should investigate BCAA metabolic pathways and their effects on milk amino acids in *Yili* mares, providing theoretical foundations and metabolic explanations for improving milk quality. This study also establishes a basis for exploring maternal-milk amino acid metabolic relationships and regulatory mechanisms.

## Data Availability

The LC-MS amino acid quantitative data of each sample in the study are deposited in the figshare repository, accession number https://doi.org/10.6084/m9.figshare.28944284.v1.
